# A Selective Tether Recruits Activated Response Regulator CheB to Its Chemoreceptor Substrate

**DOI:** 10.1128/mBio.03106-21

**Published:** 2021-11-23

**Authors:** Mingshan Li, Xianjin Xu, Xiaoqin Zou, Gerald L. Hazelbauer

**Affiliations:** a Department of Biochemistry, University of Missouri—Columbia, Columbia, Missouri, USA; b Department of Physics and Astronomy, University of Missouri—Columbia, Columbia, Missouri, USA; c Dalton Cardiovascular Research Center, University of Missouri—Columbia, Columbia, Missouri, USA; d Institute for Data Science and Informatics, University of Missouri—Columbia, Columbia, Missouri, USA; Massachusetts Institute of Technology

**Keywords:** response regulators, bacterial chemotaxis, methylesterases, protein covalent modification, sensory adaptation

## Abstract

Methylesterase/deamidase CheB is a key component of bacterial chemotaxis systems. It is also a prominent example of a two-component response regulator in which the effector domain is an enzyme. Like other response regulators, CheB is activated by phosphorylation of an aspartyl residue in its regulatory domain, creating an open conformation between its two domains. Studies of CheB in Escherichia coli and related organisms have shown that its enzymatic action is also enhanced by a pentapeptide-binding site for the enzyme at the chemoreceptor carboxyl terminus. Related carboxyl-terminal pentapeptides are found on >25,000 chemoreceptor sequences distributed across 11 bacterial phyla and many bacterial species, in which they presumably play similar roles. Yet, little is known about the interrelationship of CheB phosphorylation, pentapeptide binding, and interactions with its substrate methylesters and amides on the body of the chemoreceptor. We investigated by characterizing the binding kinetics of CheB to Nanodisc-inserted chemoreceptor dimers. The resulting kinetic and thermodynamic constants revealed a synergy between CheB phosphorylation and pentapeptide binding in which a phosphorylation mimic enhanced pentapeptide binding, and the pentapeptide served not only as a high-affinity tether for CheB but also selected the activated conformation of the enzyme. The basis of this selection was revealed by molecular modeling that predicted a pentapeptide-binding site on CheB which existed only in the open, activated enzyme. Recruitment of activated enzyme by selective tethering represents a previously unappreciated strategy for regulating response regulator action, one that may well occur in other two-component systems.

## INTRODUCTION

**FIG 1:**
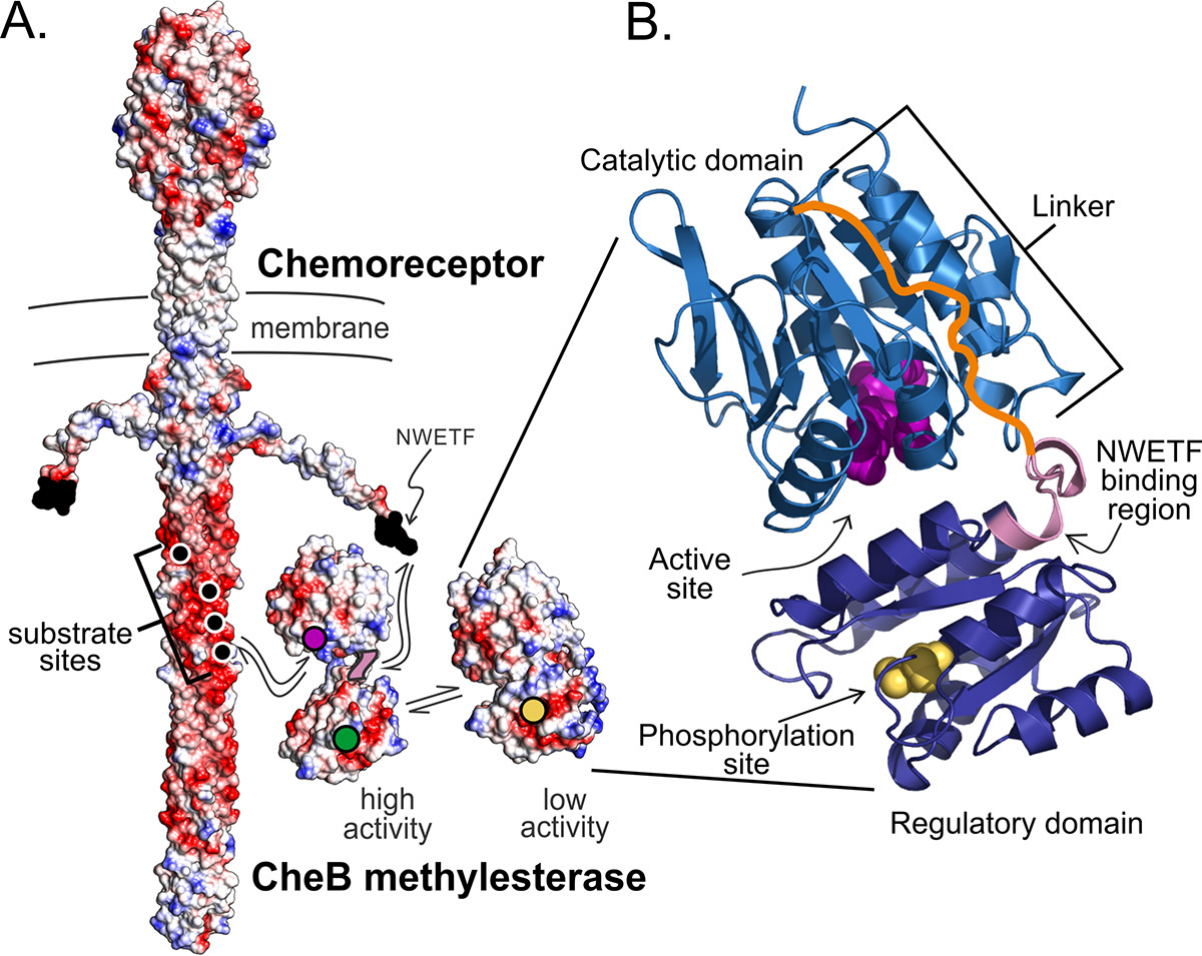
CheB methylesterase/deamidase and its chemoreceptor substrate. (A) Space-filling models of CheB and a chemoreceptor homodimer showing the interactions between the two molecules, with negatively charged amino acyl side chains in red, positively charged side chains in blue, and other side chains in white. The low-activity, closed structure of CheB (right) is from PDB accession no. 1A2O. The high-activity form (left) is a hypothetical representation of the open form (see the text). For CheB, the position of the active site is marked by a magenta circle, the positions of the NWETF pentapeptide-binding region by violet circles, and the position of the phosphoryl-accepting aspartyl side chain by a gold circle in the low-activity form and a green circle in the phosphorylated, high-activity form. The positions of the methyl-accepting sites on the chemoreceptor are marked by black circles with white borders and the positions of the CheB-binding pentapeptide are indicated as black side chains at the carboxyl termini of the carboxyl-terminal flexible arm. (B) Ribbon representation of the structure of low-activity, closed CheB (PDB accession no. 1A2O) with labeling of the two domains, the linker between them (orange), active site residues (magenta), the phosphorylation site (gold), and the pentapeptide-binding region (violet).

Throughout bacterial diversity, methylesterase/deamidase CheB is a key component in the machinery of bacterial chemotaxis (1–3). It is also a prominent example of a two-component response regulator in which the effector domain is an enzyme (4). It is one of two enzymes that covalently modify bacterial chemoreceptors. It hydrolyzes glutamyl methyl esters formed on chemoreceptors at specific methyl-accepting glutamyl resides by the action of methyltransferase CheR and creates some of the methyl-accepting sites by deamidation of glutaminyl side chains. CheB and CheR adjust the extent of receptor modification to match receptor ligand occupancy and in doing so mediate chemotactic sensory adaptation and the sensing of temporal gradients of attractants and repellents (5). Like other response regulators, CheB is activated by phosphorylation of an aspartyl residue on its regulatory domain (6–8). In Escherichia coli and related organisms, the action of CheB is also enhanced by a pentapeptide, NWETF, at the carboxyl terminus of some of its chemoreceptors (Fig. 1A) (9). The sequence is connected to the receptor body by a 35-residue unstructured flexible arm (10). The pentapeptide binds CheB (11, 12) and, when at its native location at the end of the flexible arm, enhances hydrolysis of chemoreceptor methylesters by the enzyme (13, 14). Thus, CheB binds not only to substrate side chains on the helical coil of chemoreceptor cytoplasmic domains but also to the carboxyl-terminal pentapeptide. Its action is enhanced not only by phosphorylation but also by the chemoreceptor pentapeptide. In this study, we investigated the interplay among these features. Related carboxyl-terminal pentapeptides are found on ∼11% chemoreceptor sequences (>25,000 at the time of the analysis), distributed among many bacterial species and 11 bacterial phyla (15). These carboxyl-terminal sequences presumably bind to cognate CheBs, an interaction that has been documented in the only other species investigated besides E. coli (16).

CheB is a two-domain protein, with an amino-terminal response regulator domain attached by a linker sequence to a carboxyl-terminal catalytic domain (17) (Fig. 1B). Phosphorylation of the response regulator domain by the chemotaxis histidine kinase creates a high-activity form of the methylesterase (6), shifting interaction between the two domains to an “open” conformation that relieves occlusion of the active site by the regulatory amino-terminal domain (18, 19). The enzyme binds its substrate chemoreceptor side chains with the surfaces surrounding its active site (20). In contrast, CheB binds the pentapeptide at the junction of the regulatory domain and the linker, a location removed from the active site (Fig. 1B) (11). These interactions are incompletely defined. The low-activity, i.e., unphosphorylated, form of CheB from E. coli or Pseudomonas aeruginosa binds to the pentapeptide with weak dissociation constants of 130 to 160 μM (21) and ∼90 μM (16), respectively, implying that the pentapeptide could not serve as an effective tether to increase the local concentration of the enzyme. Binding of high-activity, phosphorylated CheB to the pentapeptide has not been measured. In addition, binding to substrate sites on the coiled-coil body of a chemoreceptor has not been characterized for either CheB form but must be significantly weaker than binding to the pentapeptide because it was not detectable by assays that detected binding to the pentapeptide (21). There is no information about how activation of CheB by phosphorylation affects interaction with the pentapeptide or about the binding kinetics of either the low-activity or high-activity form of CheB to either of its sites of interaction with chemoreceptors. The current study was undertaken to provide this information. It led to new insights into the interactions of methylesterase/deamidase with chemoreceptors and identification of a previously unappreciated role for the pentapeptide.

## RESULTS

### Experimental design.

We determined the kinetics of association and dissociation of methylesterase/deamidase CheB in its low- and high-activity form with the E. coli aspartate chemoreceptor Tar using biolayer interferometry (22, 23) and isolated receptor homodimers in Nanodiscs (24, 25). Apparent rate constants of association and dissociation were derived from global fits of time courses for binding and release (see Fig. S1 in the supplemental material). Equilibrium dissociation constants were calculated from those apparent rate constants.

10.1128/mBio.03106-21.1 FIG S1 Example time courses of CheB binding to Tar. Time courses from five consecutive runs of CheB at different concentrations binding to immobilized Tar4E homodimers. For each time course, a sensor tip decorated with avidin to which were bound biotinylated Tar homodimers in Nanodiscs was placed in buffer and a baseline was established (240 to 255 s). The tip was then placed in a tube containing a solution of CheB at a specific concentration. After 30 s, at which time binding of CheB and Tar was close to an apparent equilibrium, the tip was switched to a tube of buffer, and dissociation was monitored. The global fit to the data is shown as a thin black line. Download FIG S1, TIF file, 1.9 MB. Copyright © 2021 Li et al. 2021 Li et al. https://creativecommons.org/licenses/by/4.0/ This content is distributed under the terms of the Creative Commons Attribution 4.0 International license.

### CheB interactions with the NWETF.

We measured binding of the low-activity, unphosphorylated form of CheB to the NWETF pentapeptide tethered to the sensor surface via a linker or in its native position at the carboxyl terminus of Tar4E or Tar4Q (23). Equilibrium dissociation constants were essentially the same for all conditions, 190 ± 85 μM for pentapeptide alone, and 230 ± 70 and 160 ± 30 for the pentapeptide on Tar4E or Tar4Q, respectively (Table 1). Within the error of the measurements, these values corresponded to those previously determined for free pentapeptide binding to unphosphorylated CheB by isothermal titration calorimetry (160 μM) and equilibrium dialysis (130 μM) and for pentapeptide at the carboxyl terminus of TarQEQE in native membrane binding unphosphorylated CheB determined by a pulldown assay (∼150 μM equilibrium dissociation constant [*K_D_*]) (21). These results validated our procedure for measuring CheB binding and indicated that binding of the enzyme to pentapeptide-bearing Tar is essentially binding to its pentapeptide. In addition, they showed that the signaling state of the chemoreceptor coiled-coil body did not influence the binding of its pentapeptide to low-activity CheB, consistent with its separation from the receptor coiled-coil body by an unstructured flexible arm (10).

**TABLE 1:**
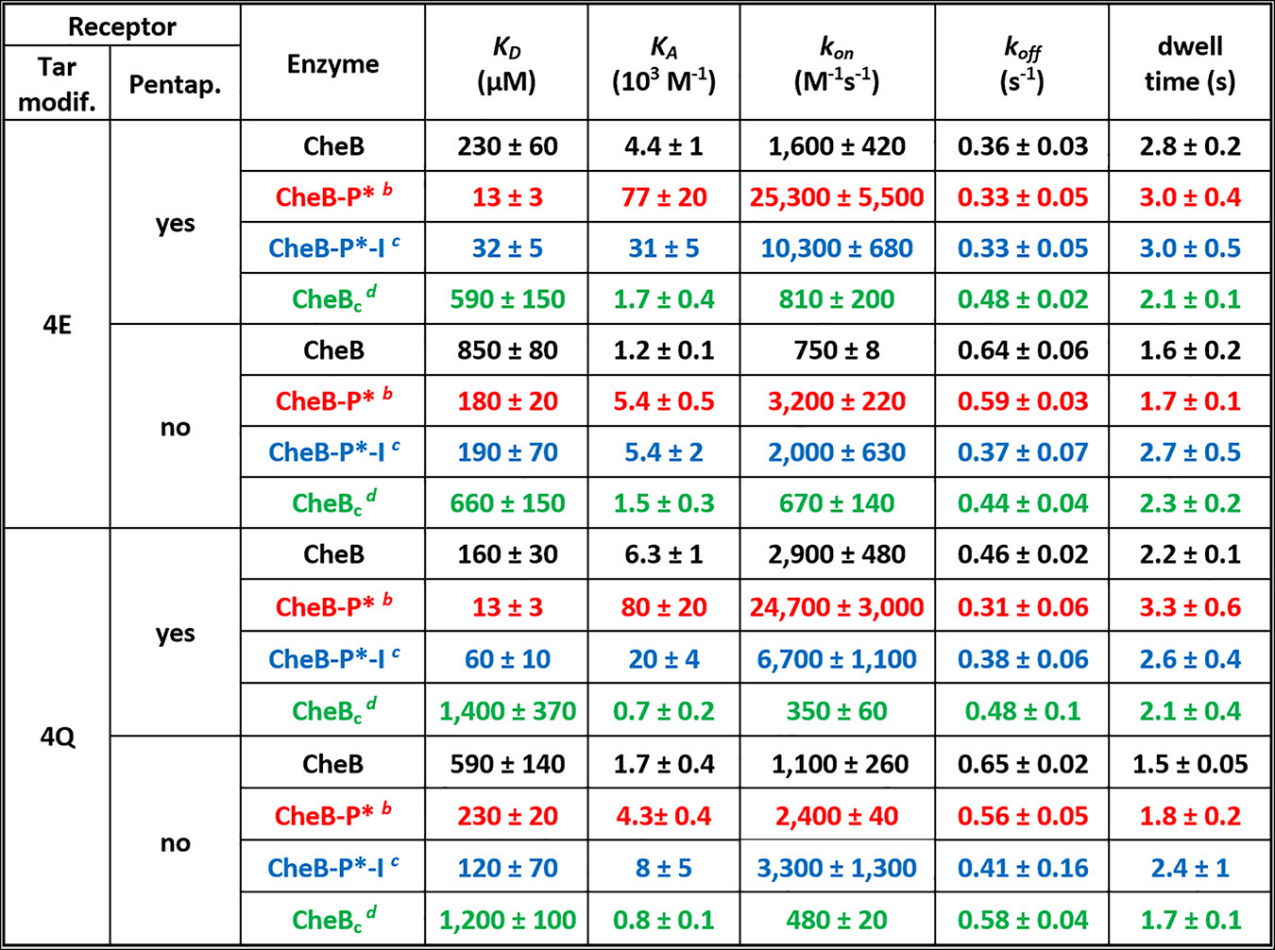
Kinetic and thermodynamic parameters of CheB binding^*a*^

a Values for *k*_on_ and *k*_off_ are averages from at least three independent experiments with standard deviations. Values for *K_D_*, *K_A_*, and dwell time were calculated from the mean values for *k*_on_ and *k*_off_ with standard deviations calculated as the respective propagated error. Tar modif., Tar modification state; Pentap., pentapeptide.

b Synthetic phosphorylation using SPO_3_^−2^ coupled to Cys at phosphorylation site to create synthetically activated enzyme (26).

c Synthetically phosphorylated enzyme carrying active site mutation S164A.

d Catalytic domain of CheB which lacks the regulatory domain and linker.

**FIG 2:**
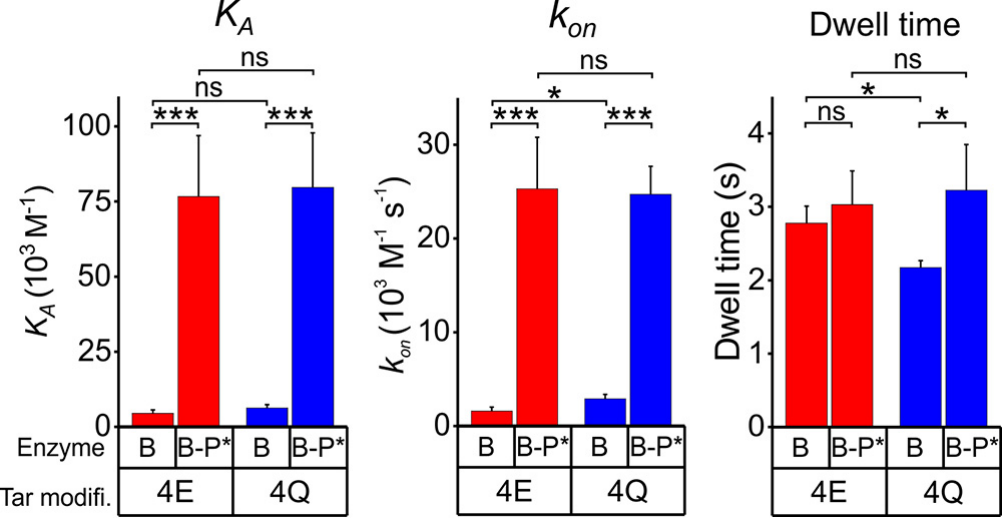
Parameters for binding of forms of the methylesterase/deamidase to pentapeptide-bearing chemoreceptor Tar. The panels compare the equilibrium association constants, *K_A_* (left), association rate constants, *k*_on_ (center) and dwell times, the inverse of the dissociation rate constant *k*_off_, (right) for binding of the low-activity, unmodified form of CheB (B) and the high-activity, phosphorylation-mimic form (B-P*) to intact (pentapeptide bearing) Tar homodimers in the all-glutamyl (4E) or all-glutaminyl (4Q) forms inserted in Nanodisc-enclosed native lipid bilayers. The error bars are standard deviations from the mean for at least three independent measurements. Numerical values for the parameters and errors are shown in Table 1. Statistical significance between pairs of conditions was calculated using a two-sample, *t* test assuming equal variance (*, *P* < 0.05; ***, *P* < 0.001; ns, not significant [*P* ≥ 0.05]). Tar modifi., Tar modification state.

A high-activity form of the methylesterase/deamidase, designated CheB-P, is created by phosphorylation at a specific aspartyl residue in its N-terminal regulatory domain (6–8). However, activation is short-lived because of a high rate of autodephosphorylation (7), making it difficult to investigate binding of this activated form of the enzyme to chemoreceptors with confidence. Instead, we used a stable, functionally active mimic of phosphorylated CheB from Salmonella enterica subsp. *enterica* serovar Typhimurium (hereafter Salmonella) characterized by Saxl and coworkers (26). In this synthetic mimic, a cysteinyl residue substituted for the native, phosphoryl-accepting aspartyl residue is modified by sodium thiophosphate to introduce a stable negative charge at the site of phosphorylation and thus activate the enzyme to essentially the same extent as native phosphorylation (26). We measured rates of association and dissociation of this activated mimic, CheB-P*, for pentapeptide-bearing Tar4Q and Tar4E. To best illustrate enhancements in enzyme-receptor interactions generated by phosphorylation, Fig. 2 displays parameters that increase with increasing affinity. Thus, the figure compares equilibrium association constants *K_A_*s, the inverse of *K_D_*s, and dwell times, the inverse of dissociation rate constants, as well as association rate constants. Equilibrium dissociation constants for CheB-P* bound to pentapeptide-bearing receptor were 13 ± 3 μM, 12- to 18-fold tighter than low-activity CheB (Table 1). The stronger binding reflected substantial increases in association rate constants (Fig. 2; Table 1). As was the case for low-activity CheB, kinetic and equilibrium values for high-activity CheB-P* were not significantly affected by the signaling state of the receptor (Fig. 2; Table 1). As will be considered in detail below, stronger binding to pentapeptide-bearing chemoreceptors of high-activity CheB-P* versus low-activity CheB can be understood as the consequence of the availability of the pentapeptide-binding site only on the open, active conformation of the enzyme.

### CheB-receptor interactions in the absence of the NWETF pentapeptide.

**FIG 3:**
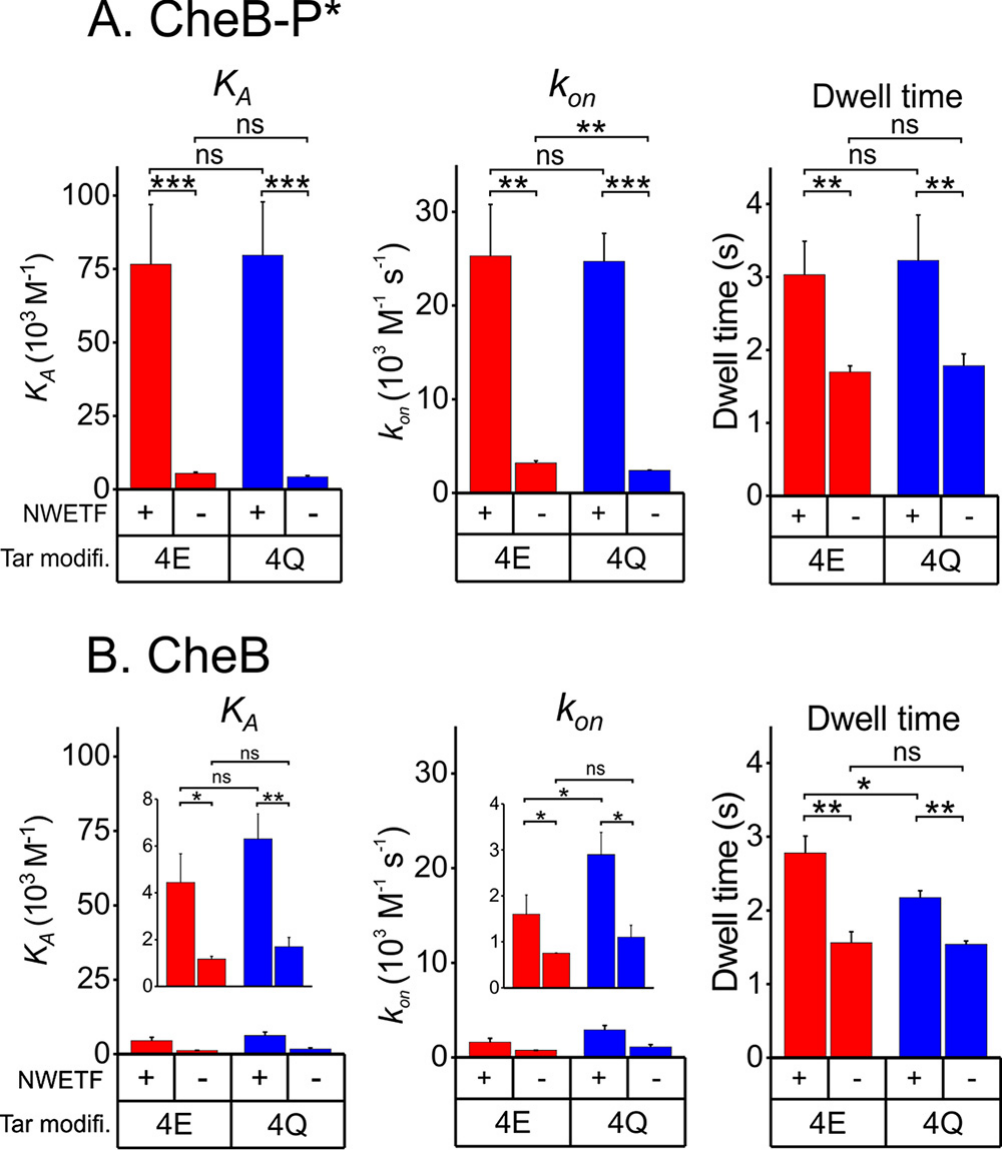
Parameters for binding of forms of methylesterase/deamidase to chemoreceptor Tar bearing or lacking the pentapeptide. The panels compare the equilibrium association constants, *K_A_* (left), association rate constants, *k*_on_ (center), and dwell times, 1/*k*_off_ (right) for binding of the high-activity, phosphorylation-mimic form of CheB (A) or its low-activity, unmodified form (B) to Tar homodimers with pentapeptide (NWETF+) or deleted of that carboxyl-terminal sequence (NWETF-) in the all-glutamyl (4E) or all-glutaminyl) forms inserted in Nanodisc-enclosed native lipid bilayers. Data are plotted on the same scale as other figures for easy comparison with those figures and where appropriate in expanded-scale insets. The error bars are standard deviations from the means for at least three independent measurements. Numerical values for the parameters and errors are shown in Table 1. Statistical significance between pairs of conditions was calculated using a two-sample, *t* test assuming equal variance (*, *P* < 0.05; **, *P* < 0.01; ***, *P* < 0.001; ns, not significant [*P* ≥ 0.05]). Tar modifi., Tar modification state.

It had not been possible by standard methods to detect the weak binding of the methylesterase/deamidase to a chemoreceptor lacking the NWETF pentapeptide (9, 21). Detecting such weak interactions required an assay that could be performed at the high protein concentrations necessary to detect low-affinity binding. Biolayer interferometry met that requirement (22, 23). Thus, we were able to measure interactions of low-activity CheB and high-activity CheB-P* with the coiled-coil chemoreceptor body. We determined kinetic parameters and calculated equilibrium dissociation constants for TarΔpp, which lacks the carboxyl-terminal pentapeptide, in the two extremes of modification, the substrate form Tar4QΔpp and product form, Tar4EΔpp. Interactions of each form with its substrate and product sites on the chemoreceptor body were weaker than the respective interactions with pentapeptide-containing receptor (Fig. 3; Table 1). High-activity CheB-P* bound to either form of Tar4Δpp with a *K_D_* of ∼200 μM, 15-fold weaker than to pentapeptide (Fig. 3A). Binding of CheB-P* to substrate and product sites on the receptor body was distinctly different than binding to the pentapeptide, with apparent association rate constants (*k*_on_s) approximately ninefold lower and dwell times almost twofold lower (Fig. 3A; Table 1). There was no statistically significant preference for binding to the substrate (4Q) or product (4E) form of the chemoreceptor (Fig. 3A; Table 1). Low-activity CheB bound to its substrate and product sites even more weakly with *K_D_*s of 590 ± 140 μM to Tar4QΔpp and 850 ± 80 μM to Tar4EΔpp (Fig. 3B; Table 1), values not significantly different (Fig. 3B). Those affinities were approximately fourfold weaker than the respective binding to pentapeptide-bearing receptors, with differences in *k*_on_ and dwell time (Fig. 3B; Table 1). Interactions with substrate and product forms of the chemoreceptor body of low-activity and high-activity forms of CheB are compared in Fig. S2.

10.1128/mBio.03106-21.2 FIG S2 Parameters for binding of forms of the methylesterase/deamidase to chemoreceptor Tar lacking the pentapeptide. The panels compare the equilibrium association constants, *K_A_* (left), association rate constants, *k*_on_ (center) and dwell times, the inverse of the dissociation rate constant *k*_off_, (right) for binding of the low-activity, unmodified form of CheB (B) and the high-activity, phosphorylation mimic form (B-P*) to TarΔpp homodimers in the all-glutamyl (4E) or all-glutaminyl (4Q) forms inserted in Nanodisc-enclosed native lipid bilayers. Data are plotted on the same scale as other figures for easy comparison with those figures and where appropriate in expanded-scale insets. The error bars are standard deviations from the means for at least three independent measurements. Numerical values for the parameters and errors are shown in Table 1. Statistical significance between pairs of conditions was calculated using a two-sample, *t* test assuming equal variance (*, *P* < 0.05; **, *P* < 0.01; ***, *P* < 0.001; ns, not significant [*P* ≥0.05]). Download FIG S2, TIF file, 2.6 MB. Copyright © 2021 Li et al. 2021 Li et al. https://creativecommons.org/licenses/by/4.0/ This content is distributed under the terms of the Creative Commons Attribution 4.0 International license.

**FIG 4:**
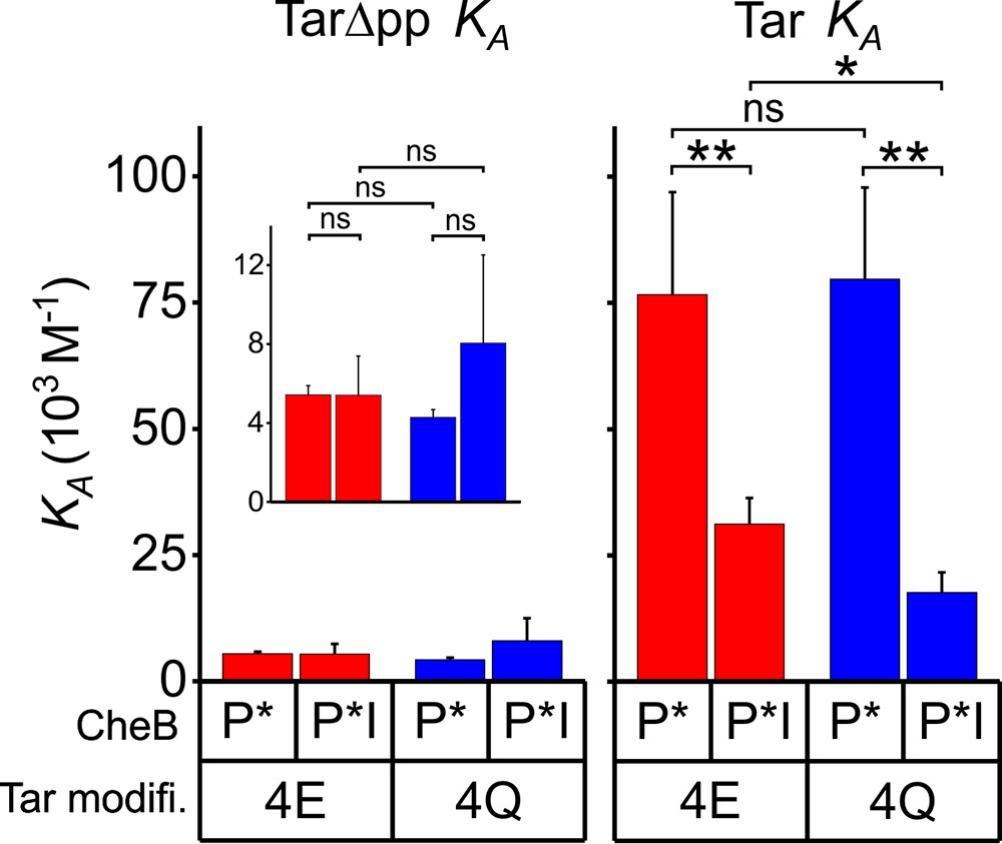
Parameters for binding of the phosphorylation mimic and the enzymatically inactive phosphorylation mimic of the methylesterase/deamidase to forms of chemoreceptor Tar. The panels compare the equilibrium association constants *K_A_* for binding of the high-activity, phosphorylation-mimic form (P*) and the phosphorylation-mimic containing the enzymatically inactivating mutational substitution S164A (P*-I) to Tar homodimers lacking the pentapeptide (left panel) or carrying that sequence (right panel) in the all-glutamyl (4E) or all-glutaminyl (4Q) forms inserted in Nanodisc-enclosed native lipid bilayers. Data are plotted on the same scale as other figures for easy comparison with those figures and where appropriate in expanded-scale insets. The error bars are standard deviations from the means for at least three independent measurements. Numerical values for the parameters and errors are shown in Table 1. Statistical significance between pairs of conditions was calculated using a two-sample, *t* test assuming equal variance (*, *P* < 0.05; **, *P* < 0.01; ***, *P* < 0.001; ns, not significant [*P* ≥ 0.05]). Tar modifi., Tar modification state.

It was possible that high-activity CheB-P* deamidated Tar4QΔpp sufficiently during the binding assay to obscure interactions with the substrate form of the receptor. Thus, we tested binding by an enzymatically inactive form CheB-P*-I, which carried the phosphorylation mimic but also had the catalytic serine replaced by alanine. Within the error of the measurements, the inactivating mutation did not alter the equilibrium binding constant for Tar4QΔpp or Tar4EΔpp (Fig. 4, left panel), although there were modest and in large part compensatory changes in association rate constants and dwell times (Table 1). We conclude that the altered catalytic side chain is not a major contributor to the energy of binding to substrate and product sites on the receptor body and that any enzymatic deamidation occurring over the few minutes of binding measurements did not significantly influence determinations of affinity of activated enzyme for the substrate form of the receptor. In contrast, substitution of the catalytically crucial serine with alanine reduced ∼2.5-fold the affinity (*K_A_*) of the enzyme for either modification state of pentapeptide-bearing Tar (Fig. 4, right panel). The reductions were the result of reduced *k*_on_s with no statistically significant effect on dwell times (Table 1). Reductions in *k*_on_s and correspondingly *K_A_*s can be understood as a consequence of the serine-to-alanine substitution at the interface between the two CheB domains, shifting the conformational equilibrium toward the closed state and thus reducing the proportion of enzyme in the open conformation in which the pentapeptide-binding site is available.

### Interactions of receptor and CheB lacking the regulatory domain and linker.

CheB can be activated by deletion of the regulatory domain (6, 27), albeit not as effectively as by phosphorylation (8). To investigate the role binding affinity might have in that activation, we measured binding of CheB_C_, a form of the methylesterase lacking the amino-terminal regulatory domain and linker, to pentapeptide-bearing and pentapeptide-lacking forms of Tar. This truncated protein bound much more weakly to chemoreceptors than the activated phosphorylation mimic CheB-P* but exhibited an approximately twofold preference for the product form Tar4E (*K_D_* of 660 ± 150 μM) versus the substrate form Tar4Q (1,200 ± 100 μM) (Fig. S3; Table 1). There was no significant difference in binding in the presence or absence of pentapeptide (Table 1), as would be expected for the response regulator missing the pentapeptide-binding site. We conclude that activation of CheB enzymatic activity by deletion of the regulatory domain is not the result of enhanced receptor binding.

10.1128/mBio.03106-21.3 FIG S3 Parameters for binding of the catalytic domain of methylesterase/deamidase to chemoreceptor Tar carrying or lacking the pentapeptide. The panels compare the equilibrium association constants, *K_A_* (left), association rate constants, *k*_on_ (center), and dwell times, the inverse of the dissociation rate constant *k*_off_, (right) for binding of CheB_C_ to Tar homodimers with pentapeptide (NWETF +) or deleted of that carboxyl-terminal sequence (NWETF -) in the all-glutamyl (4E) or all-glutaminyl (4Q) form inserted in Nanodisc-enclosed native lipid bilayers. Data are plotted on the same scale as other figures for easy comparison with those figures and where appropriate in expanded-scale insets. The error bars are standard deviations from the meand for at least three independent measurements. Numerical values for the parameters and errors are shown in Table 1. Statistical significance between pairs of conditions was calculated using a two-sample, *t* test assuming equal variance (*, *P* < 0.05; **, *P* < 0.01; ***, *P* < 0.001; ns, not significant [*P* ≥0.05]). Download FIG S3, TIF file, 2.5 MB. Copyright © 2021 Li et al. 2021 Li et al. https://creativecommons.org/licenses/by/4.0/ This content is distributed under the terms of the Creative Commons Attribution 4.0 International license.

### Modeling the CheB pentapeptide-binding site.

**FIG 5:**
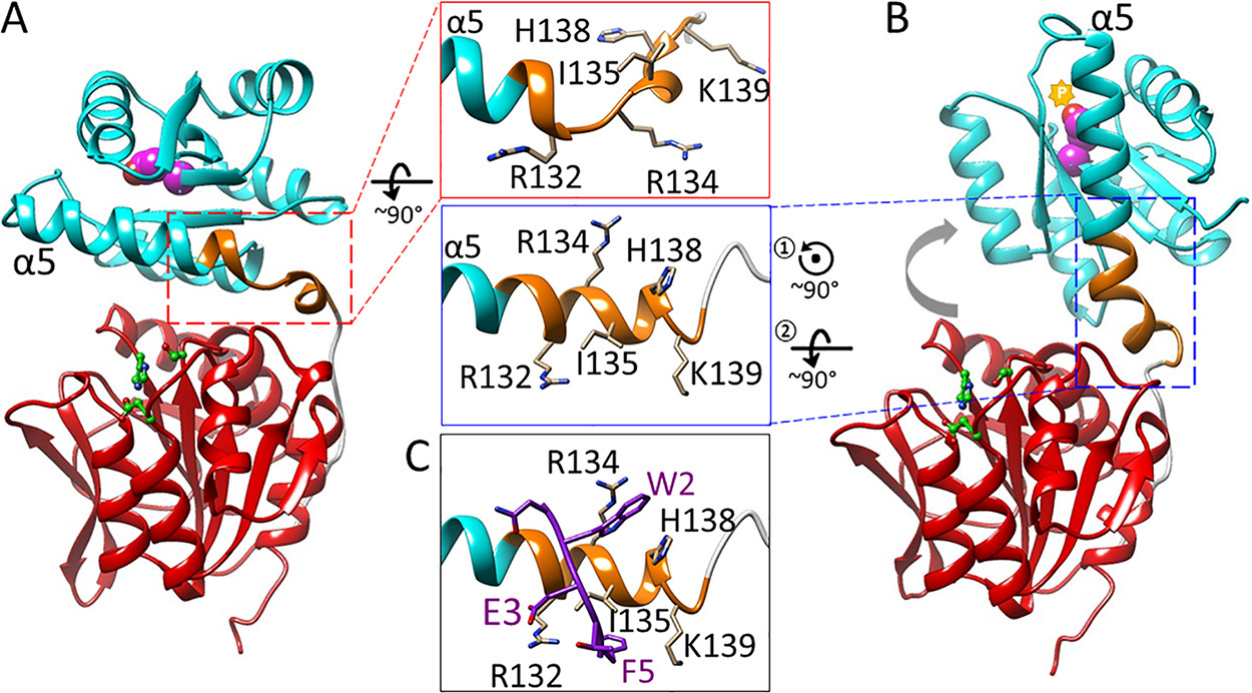
Model of the open, high-activity form of CheB and its interaction with the pentapeptide. Ribbon representations of Salmonella CheB with the regulatory domain in blue, the catalytic domain in red, the linker in gold, the phosphorylation site as purple spheres, the active site residues as green sticks and balls, and enlargements of the region identified as containing the pentapeptide-binding site with key side chains indicated (center images). (A) The closed, low-activity conformation of CheB (PDB accession no. 1A2O). (B) The modeled open, high-activity conformation of CheB showing phosphorylation as a gold star, the extension of helix α5 (see the text), and repositioning of key residues. (C) Modeled docking of the pentapeptide (purple stick representation) on extended helix α5.

**FIG 6:**
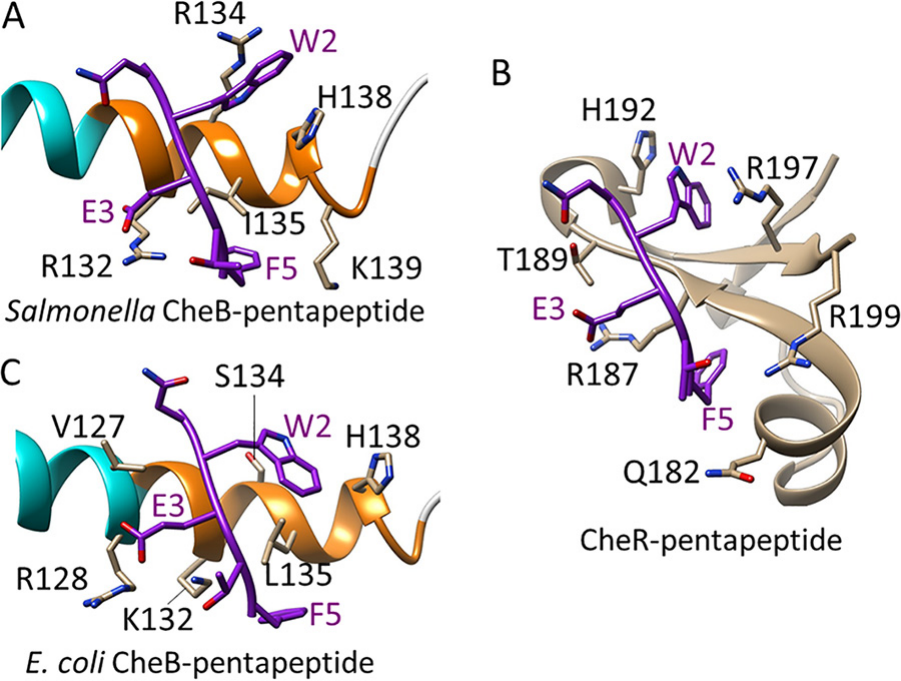
NWETF pentapeptide binding to CheB and CheR. Relevant portions of the enzymes are shown as ribbon diagrams with key side chains indicated and the bound pentapeptide in purple. (A) Modeled docking of NWETF on extended helix α5 of Salmonella CheB as shown in Fig. 5C. (B) X-ray crystallographic structure of NWETF bound to Salmonella CheR as a fourth strand to a three-strand beta sheet of the beta subdomain (17) (PDB accession no. 1BC5). (C) Modeled docking of NWETF on extended helix α5 of E. coli CheB.

The difference we observed in pentapeptide binding between unmodified CheB and its phosphorylated mimic (Fig. 2; Table 1) can be understood by postulating that the pentapeptide-binding site is available in the open but not closed conformation. That site was localized previously to an 11-residue segment at the carboxyl end of the regulatory domain and the first part of the linker (Fig. 1) (11). Specifically, a proteolytic fragment beginning near the amino terminus of α5 and extending through the catalytic domain bound the pentapeptide, but a fragment beginning at the first residue of the linker did not, pentapeptide protected intact CheB from limited proteolysis at position 132, and a peptide from CheB residues 130 through 140 reduced binding of intact enzyme to pentapeptide (11). However, the specific binding mode of pentapeptide was not determined. Since no structure was available for activated, open conformation of CheB, we turned to molecular modeling, focusing on the 11-residue segment, residues 130 through 140. Eight of those residues are in the linker between the two CheB domains. Linkers in response regulators are conformationally flexible. They can be without regular secondary structure or be partly or entirely helical in different crystallographic or functional forms (16, 28, 29). The CheB linker has little regular secondary structure in the closed form, but secondary structure prediction (30) suggested that helix α5 could extend past its closed conformation termination at residue 132 to include residues through 138 in the linker, thus incorporating what had been nonhelical residues 133 and 134 plus the single helical turn from residues 135 to 138. We modeled the open form of CheB with this extended α5 helix (Fig. 5B; see Materials and Methods for details) (31). Extended α5 was probed for pentapeptide binding using the protein-peptide docking program MDockPeP (32, 33). The top four scoring models (Fig. S4) were assessed for correspondence with previous biochemical characterization of the importance of each NWETF side chain in binding CheB (12). One model among the top four had the crucial interactions with pentapeptide side chains of W2, F5, and E3 (Fig. 5C and Fig. S4), the three residues most important for binding of CheB to pentapeptide (12). Analysis of phi/psi angles (34) for residues 130 to 139 in this modeled docking were in highly preferred conformations. To confirm the importance of the aromatic side chain interactions in the modeled docking, we calculated docking scores of peptides with alanine in place of W2, F5, or both and found substantial reductions from −67.4 for the native sequence to −43.2, −44.8, or −20.5, respectively, for the respective alanine-substituted pentapeptides. Furthermore, the way in which the pentapeptide bound to extended α5 was strikingly similar to pentapeptide binding determined by crystallography to its other ligand, methyltransferase CheR (PDB accession no. 1BC5 [35]) (Fig. 6A and B). For pentapeptide binding to both CheB and CheR from Salmonella, (i) the tryptophan side chain at pentapeptide position 2 inserts into a pocket formed by side chains of an arginine and a histidine, (ii) the negatively charged glutamyl side chain at position 3 forms a salt bridge with a positively charged arginine side chain, and (iii) phenylalanine side chain at position 5 packs in a hydrophobic pocket (Fig. S5). Importantly, this set of concerted pentapeptide interactions cannot occur with the closed unphosphorylated conformation of CheB (PDB accession no. 1A2O [17]). In that conformation, crucial packing residue H138 is largely buried in the domain interface (Fig. S6), and residues R134 and H138 are not positioned to form a pocket for the pentapeptide tryptophan. Instead, their side chains are distant and point in opposite directions (Fig. 5A, top center inset). Thus, the postulated pentapeptide-binding site does not exist in the closed state of CheB but only in the open state.

10.1128/mBio.03106-21.4 FIG S4 Top-four rated binding modes predicted for the pentapeptide binding to the extended α5 helix of Salmonella CheB. The relevant portion of the extended helix is shown as a ribbon diagram (blue for native α5 and orange for its modeled extension) with key side chains indicated and the bound pentapeptide in purple. Model 4 satisfies all biochemical information about the relative importance for binding of the side chains of the pentapeptide (see Results). Download FIG S4, TIF file, 0.8 MB. Copyright © 2021 Li et al. 2021 Li et al. https://creativecommons.org/licenses/by/4.0/ This content is distributed under the terms of the Creative Commons Attribution 4.0 International license.

10.1128/mBio.03106-21.5 FIG S5 Views of the pentapeptide packing on extended helix α5 of Salmonella CheB. The packing of the pentapeptide on extended helix α5 as in Fig. 5C is shown with surface depictions of the CheB segment and the pentapeptide represented by a stick (A) or surface (B) model. The view in panel A illustrates the positions of the two aromatic side chains on the pentapeptide: W2 (on the right) and F5 (on the left). The view in panel B illustrates the stacking of pentapeptide W2 between the aliphatic portion of CheB R134 and the ring of CheB H138. Download FIG S5, TIF file, 2.9 MB. Copyright © 2021 Li et al. 2021 Li et al. https://creativecommons.org/licenses/by/4.0/ This content is distributed under the terms of the Creative Commons Attribution 4.0 International license.

10.1128/mBio.03106-21.6 FIG S6 Key pentapeptide-binding residue H138 is largely buried in the low-activity, closed conformation of CheB. Surface depictions of the X-ray structure of Salmonella CheB in its low-activity, closed conformation (PDB accession no. 1A2O) illustrate the limited accessibility of the H138 side chain. H138 is in red; the rest of CheB in blue. Download FIG S6, TIF file, 2.1 MB. Copyright © 2021 Li et al. 2021 Li et al. https://creativecommons.org/licenses/by/4.0/ This content is distributed under the terms of the Creative Commons Attribution 4.0 International license.

Modeling pentapeptide binding to CheB from E. coli revealed plasticity in the binding mode. The pentapeptide-binding region of E. coli CheB differs from Salmonella CheB at three positions, including one directly involved in binding. However, pentapeptide docked effectively on the extended α5 helix of E. coli CheB (Fig. 6C). The side chain of pentapeptide residue F5 packed in a location and orientation similar to that observed for Salmonella CheB. However, there was an amino-terminal shift in the position of pentapeptide on extended α5 of E. coli CheB, and the other two crucial side chain interactions were different from those observed for Salmonella CheB (compare Fig. 6A and C). The shift resulted in the negatively charged side chain of E3 forming a salt bridge with the CheB R128 side chain, rather than R132 as seen for docking to the Salmonella sequence. In addition, in E. coli CheB, position 134 is a serine, not an arginine as in Salmonella, and thus, there was not a pocket into which the W2 side chain could insert. Instead, the W2 side chain packed on the surface of the helix, between residues L135 and H138, creating T-shaped Pi stacking with the histidine. Thus, it appears that the extended α5 helix of CheB provides an effective platform for pentapeptide binding that accommodates side chain variation. This plasticity implies that there may be considerable variation among CheB pentapeptide-binding sites in other species.

**FIG 7:**
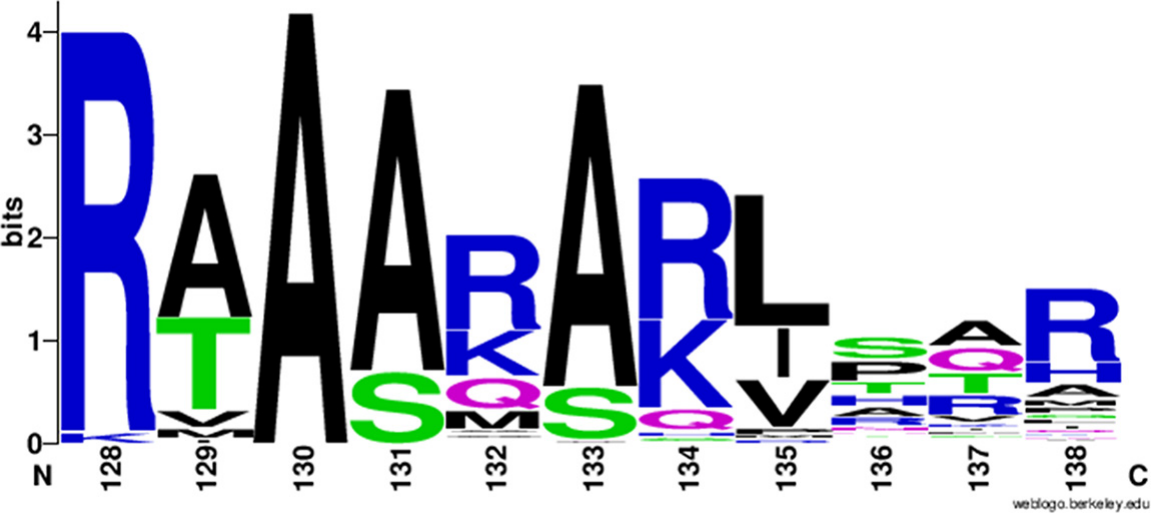
Sequence logo of the modeled pentapeptide-binding region generated from alignment of nonredundant (threshold 96%) CheB sequences with identities of >30% to Salmonella CheB from 107 bacterial species with a single CheB gene and at least one chemoreceptor gene coding for a carboxyl-terminal NWETF or NWESF. Table S1 in the supplemental material shows the sequence alignment from which the logo is derived.

We explored this issue by assessing conservation of key residues in CheBs from other bacterial species. We identified 107 containing at least one chemoreceptor with a carboxyl-terminal NWET/SF pentapeptide and a single CheB (see Table S1 in the supplemental material). As illustrated by a sequence logo of CheB residues corresponding to Salmonella CheB positions 128 through 138 (Fig. 7), there was significant conservation of identity or similarity of key binding residues identified for Salmonella or E. coli CheB. Specifically, there was a predominance of positively charged side chains at positions 128, 132, and 134, hydrophobic side chains at position 135, and large side chains at position 138. We conclude that pentapeptide-binding sites we identified in Salmonella and E. coli CheB are significantly conserved, at least in related organisms.

10.1128/mBio.03106-21.7 TABLE S1 Alignment of nonredundant CheB sequences from bacterial species containing 1 CheB and ≥1 chemoreceptor with a carboxyl-terminal pentapeptide NWETF or NWESF. Download Table S1, DOCX file, 1.9 MB. Copyright © 2021 Li et al. 2021 Li et al. https://creativecommons.org/licenses/by/4.0/ This content is distributed under the terms of the Creative Commons Attribution 4.0 International license.

## DISCUSSION

Characterization of binding of the methylesterase/deamidase CheB provided new information and insights into interactions of this key chemotaxis enzyme with the chemoreceptors it modifies. Notably, our studies revealed a crucial synergy between enzyme phosphorylation and binding to the pentapeptide tether on the chemoreceptor carboxyl terminus, thereby documenting a previously unappreciated mechanism for controlling the action of a response regulator. Our quantitative measurements of methylesterase/deamidase binding provided a comprehensive view of the multiple interactions of this two-component enzyme, including establishing that the carboxyl-terminal pentapeptide serves as a high-affinity tether for the activated methylesterase. The measurements showed that differences in affinity of high- and low-activity CheB were primarily differences in rate constants of association, not dissociation, suggesting that the two differed in the balance between open and closed forms of the two-component enzyme.

### CheB interactions with its chemoreceptor substrate.

The data summarized in Table 1, Fig. 2 to 4, and Fig. S2 and S3 in the supplemental material provide a comprehensive picture of interactions of CheB with its chemoreceptor substrate. Key to interpreting the data is the notion that the enzyme is in equilibrium between a closed, enzymatically inactive conformation and an open, enzymatically active conformation and that the equilibrium is shifted from predominately closed to predominately open by phosphorylation (8). The differences observed in pentapeptide binding between unmodified CheB and its phosphorylated mimic can be understood as reflecting the absence of the pentapeptide-binding site in the closed conformation but its presence in the open conformation. Thus, the apparently low-affinity binding of pentapeptide by unmodified, low-activity CheB would be the result of a small proportion of unmodified enzyme in the open, binding-competent state. This notion is supported by comparison of kinetic parameters for pentapeptide binding by unmodified, low-activity and phosphorylated, high-activity forms of the enzyme. There is little difference between the forms in dissociation rate constants (and thus corresponding dwell times), but there is a major difference in association rate constants (Fig. 2; Table 1). If only the open form binds pentapeptide, then dissociation rate constants and corresponding dwell times determined for unmodified and phosphorylated populations of CheB should reflect dissociation of the same complex and thus be similar. This is exactly what we observed. If only the small proportion of unmodified CheB is in the active, binding-competent conformation, then the association rate constant, calculated using total enzyme concentration, would be significantly lower than for phosphorylated enzyme, for which a much larger proportion is in the binding-competent conformation. Correspondingly, the apparent equilibrium binding constant would appear much weaker for unmodified CheB, as it does (Fig. 3; Table 1). If phosphorylated CheB were 100% open, the proportion of unmodified enzyme in the open state would correspond to the ratio of apparent equilibrium binding constants for the two forms or ∼7% of the unmodified enzyme in the open state (Table 1).

Our data identified the pentapeptide as a high-affinity tether for the open form of CheB, as had been previously shown for CheR (36). The association rate constant for binding of CheB-P* to receptor-borne pentapeptide is approximately 10-fold higher than the corresponding constant for binding to the receptor body (Table 1). Thus, in large part, the activated enzyme would bind first to pentapeptide and only subsequently to substrate and product sites on the receptor body. That subsequent binding would occur with high probability because tethering by pentapeptide would increase the local concentration of active CheB to over 1,000 μM (23), approximately fivefold above the equilibrium dissociation constant. The ∼3-s dwell times on the pentapeptide would allow sufficient time for diffusional collision of the tethered enzyme to a substrate site on the receptor body.

### Synergy between phosphorylation and pentapeptide binding.

The notion that only the open CheB has the pentapeptide-binding site is strongly supported by our modeling of the binding side region and of its binding to pentapeptide (Fig. 5). Specifically, the modeled pentapeptide-binding site does not exist in the closed conformation of CheB. A prominent feature of that site is a pocket for the pentapeptide tryptophan. That tryptophan is a major contributor to the energy of interaction between the response regulator and pentapeptide (12). The pocket is formed by the side chains of His138 and Arg134. However, in the closed conformation, the histidine side chain of residue 138 is substantially buried in the interface between the regulatory and catalytic domains and thus not available to be part of the tryptophan-binding pocket (Fig. S6). In addition, in the closed conformation, the side chains of His138 and Arg134 are distant from each other and point in opposite directions (Fig. 5, top center panel). Thus, the modeled pentapeptide-binding site does not exist in the closed conformation of CheB. Instead, it is formed by the postulated helical extension that is part of the conformational change from closed to open.

Notably, formation of the pentapeptide-binding site in the transition to the open conformation involves a change in secondary structure for only two residues, at positions 133 and 134. In the structure of the closed conformation (PDB accession no. 1A2O), those two positions are not helical but connect the carboxyl terminus of helix α5 of the regulatory domain and the amino terminus of the four-residue helical turn from positions 135 through 138 in the linker (Fig. 5). In the modeled open conformation, positions 133 and 134 assume helical secondary structure, thus extending helix α5 through position 138 and orienting the residues of this extended helix to provide the pentapeptide-binding site. This low-energy conformational change could easily be accommodated as part of the more energetically demanding changes in domain interactions driven by phosphorylation, providing an elegant mechanism for creating the pentapeptide-binding site in the open conformation. It is possible that bound pentapeptide helps stabilize the open conformation. Consistent with this notion, mutational substitutions in and near the pentapeptide-binding site shift the conformational equilibrium of unphosphorylated CheB slightly toward the open, active conformation (8), indicating that perturbing the region influences the open-closed equilibrium.

Taken together, kinetic and modeling data make a compelling case that the pentapeptide binds only to the open form of CheB, not the closed form. This implies that the pentapeptide at the carboxyl terminus of a chemoreceptor in essence selects the open, enzymatically active conformation of CheB from the population of closed and open molecules. This selective binding ensures that only enzymatically active response regulator is tethered in proximity to its substrate sites on the chemoreceptor coiled-coil body, and thus poised to modify those sites. We characterized this selective binding *in vitro*, but *in vivo* CheB localization in chemoreceptor complexes is phosphorylation dependent, implying that the same selection occurs in the cell (37).

A common feature of many signaling systems is “recruitment” of signaling proteins into complexes by specific interaction with another protein in the complex. Our characterization of interactions of CheB and receptor-borne pentapeptide provides an example of such recruitment in a bacterial chemotaxis system. CheB recruitment has the important additional feature of being specific for activated protein. We suggest other chemoreceptors carrying related carboxyl-terminal sequences perform analogous selective tethering of their cognate CheB. Furthermore, selective tethering of activated response regulators may well be a feature of two-component signaling systems in addition to those that mediate chemotaxis. More widely, selection of activated conformations of signaling proteins by selective tethers could be an unappreciated aspect of many signaling systems.

## MATERIALS AND METHODS

### Strains and plasmids.

The host for plasmids carrying *cheB* or a chemoreceptor gene was E. coli K-12 strain RP3098 (38), which produces neither chemoreceptors nor Che proteins. Genes coding for Tar4E L378C, Tar4Q L378C, Tar4EΔpp-6H L378C, and Tar4QΔpp-6H L378C were carried on plasmids pML42, pML46, pML36, and pML44, respectively (23). pCW/*cheB*, carrying E. coli *cheB* under the control of tandem *tac* promoters (39) was from F. W. Dahlquist (University of California, Santa Barbara, Santa Barbara, CA). pAL77 is a derivative of pCW/*cheB* in which the segment of *cheB* coding for residues 2 to 152 was deleted by PCR-based mutagenesis to code for CheB_C_. A plasmid carrying the gene coding for CheB D56C/C207S/C309S from Salmonella was from Ann Stock (Rutgers - Robert Wood Johnson Medical School, Piscataway, NJ). PCR-based site-directed mutagenesis of that plasmid generated pML47, coding for Salmonella CheB D56C/S164A/C207S/C309S.

### Protein purification and manipulation.

E. coli CheB and Salmonella CheB D56C/C207S/C309S and D56C/S164A/C207S/C309S were purified as described previously (11). CheB_C_ (amino acids [aa] 153 to 349) was purified using the procedure of West et al. (40), except that Bio-Gel P-60, was not used. CheB D56C/C207S/C309S and CheB D56C/S164A/C207S/C309S were modified to create a disulfide-linked phosphoryl group on D56C following the procedure described in reference 26 but using a 10-fold molar excess of DTNB [Ellman’s reagent, 5,5′-disulfanediylbis(2-nitrobenzoic acid)] rather than 90-fold and a 50-fold molar excess Na_3_SPO_3_ rather than 1,000-fold. The extent of modification was 59% for the former and 60% for the latter determined by reversed phase chromatography essentially as described previously (26) and confirmed by Nano LC-Nanospray QTQF mass spectroscopy. Membrane scaffold protein MSP1D1(-) (41) was purified as described previously (25). Tar4E L378C, Tar4Q L378C, Tar4EΔpp-6H L378C, and Tar4QΔpp-6H L378C were purified, incorporated into Nanodiscs at approximately one receptor dimer per disc, and biotinylated at L378C as described previously (23, 42). Chemoreceptor dimers in Nanodiscs exhibit native activities for ligand binding, adaptational modification, and transmembrane signaling (43, 44). These activities were confirmed for representative samples used in our analyses. Receptor-containing Nanodiscs were dialyzed into 50 mM Tris-HCl (pH 7.5), 0.5 mM EDTA, 10% (wt/vol) glycerol, and 100 mM NaCl. Prior to a binding experiment, the solution was changed using a spin desalting column to 50 mM Tris-HCl, 10% (wt/vol) glycerol, 5 mM MgCl_2_, and 50 mM KCl (pH 7.5).

### Binding assays.

Kinetics of CheB association and dissociation with Nanodisc-embedded Tar biotinylated at L378C were determined using biolayer interferometry (22, 23) as measured by a BLItz instrument (ForteBio, Menlo Park, CA, USA) and described in reference 23. Representative time course experiments are shown in Fig. S1 in the supplemental material. Measurements for CheB-P* and CheB-P*-I were in 50 mM Tris-HCl (pH 7.5), 10% (wt/vol) glycerol, 5 mM MgCl_2_, 50 mM KCl, and 2% (wt/vol) bovine serum albumin (BSA). Measurements for CheB and CheBc were in the same solution plus 2 mM dithiothreitol (DTT).

### Fitting binding data.

Using BLItz software, response curves were fit globally to a 1:1 binding model to yield apparent association rate constants (*k*_on_) and dissociation rate constants (*k*_off_). For analysis of complex patterns of binding, in which more than one binding reaction appeared to be contributing, for instance one that appeared saturable and another nonspecific, nonsaturable, the data were exported and fit globally to nonlinear exponential functions using OriginPro 2017. The best fits to the data were obtained using the following functions.

$$R=R_{0}\;+\;R_{\text{eq}}\left[a\left(e^{-k_{d1\;}t}\right)\;+\;b\left(e^{-k_{d2\;}t}\right)\right]$$

was used to globally fit dissociation time courses, with *R* being the response (in nanometers), *a + b = 1*, with *a* and *b* being the portion of the signal (in nanometers) for fast and slow phases, respectively, *k_d_*_1_ and *k_d_*_2_ being the respective dissociation rate constants, and *R*_eq_ being the response at equilibrium. Association data were fit using apparent dissociation rate constants *k_d_*_1_ and *k_d_*_2_, relevant CheB concentrations (*C*) and the equation

$$R=R_{0}\;+\;R_{\text{eq}}\left[a\left(1\;-\;e^{-(k_{a1\;}C+k_{d1})t}\right)\;+\;b\left(1\;-\;e^{-(k_{a2\;}C+k_{d2})t}\right)\right]$$

### Molecular modeling and pentapeptide docking.

MODELLER (31) was used to model the CheB regulatory domain with an extended helix α5 from residues 133 through 140, using the crystal structure of response regulator, FixJ (PDB accession no. 5XSO [28]), in which the entire linker sequence is helical and the regulatory domain has a 52% similar sequence to the CheB regulatory domain, thus facilitating superpositioning. The FixJ regulatory domain was superimposed on the regulatory domain of Salmonella CheB (PDB accession no. 1A2O) and CheB α5 extended on the template of FixJ residues 121 to 129. The remainder of the CheB regulatory domain was built using the template of its closed conformation. Figure 5B shows this modeled domain and extended linker placed in an arbitrary open position relative to the catalytic domain.

The NWETF pentapeptide was docked onto the extended α5 structure using MDockPeP (32, 33). The initial peptide conformation was extracted from the crystal structure of the pentapeptide-CheR complex (PDB accession no. 1BC5). The center of the docking box was set to the geometric center of the segment from positions 130 to 140 and its size to 25 Å to cover the entire binding region. Other parameters were default values. Constraints maintained the peptide backbone in its initial conformation and pentapeptide side chains W2, E3, and F5 within 5 Å of the segment from positions 130 to 140. Pentapeptide side chains were freely flexible. The top four models for Salmonella CheB (Fig. S4) had ITScorePeP energy scores of −80.8, −76.9, −68.3, and −67.4. Considering the indeterminacy in these scores, we considered these models equally likely.

### Sequence alignment.

The NCBI-BLAST program version 2.6.0+ (45) was used to search the UniProt database release 2021_03 (45–47) for chemoreceptors with a carboxyl-terminal NWETF or NWESF and to identify bacterial species with at least one such chemoreceptor and a single CheB. Clustering (48) and multiple sequence alignments (49) were performed for Salmonella CheB and CheBs in other species with sequence identity of >30% and a threshold of 96% to avoid redundant sequences. Table S1 in the supplemental material shows a portion of this alignment, including residues aligned with Salmonella CheB residues 132 to 138. Using this alignment, a sequence logo (Fig. 7) was generated by WebLogo (50).
