# Supplementary figures and images for: A Selective Tether Recruits Activated Response Regulator CheB to Its Chemoreceptor Substrate
Source: mBio. 2021 Nov 23;12(6):e03106-21. doi: 10.1128/mBio.03106-21 (PMC8609364; doi:10.1128/mBio.03106-21)

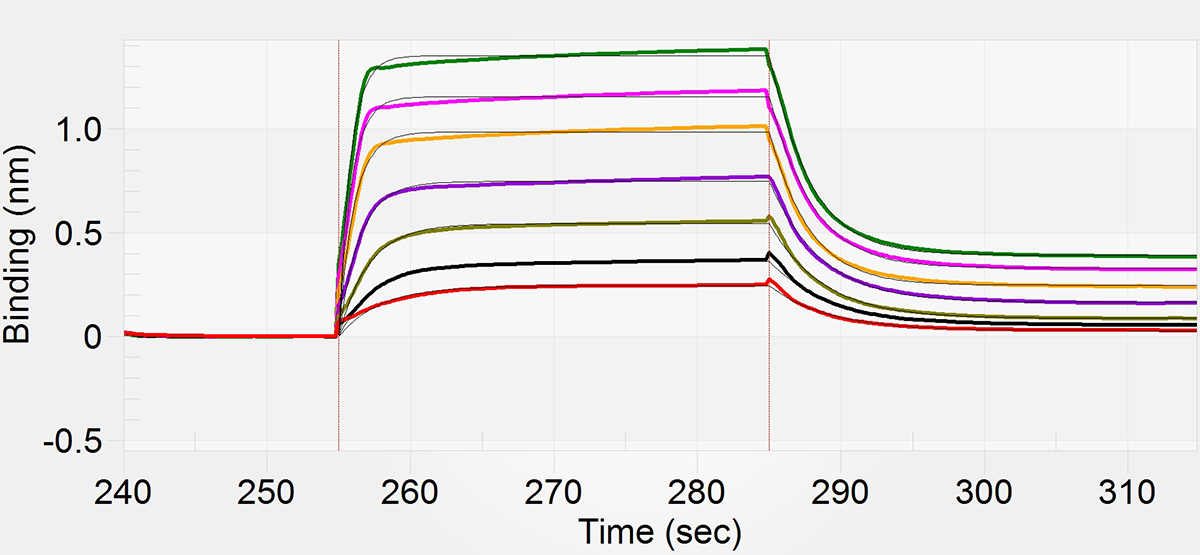

Supplement: FIG S1 [file mbio.03106-21-sf001.tif]

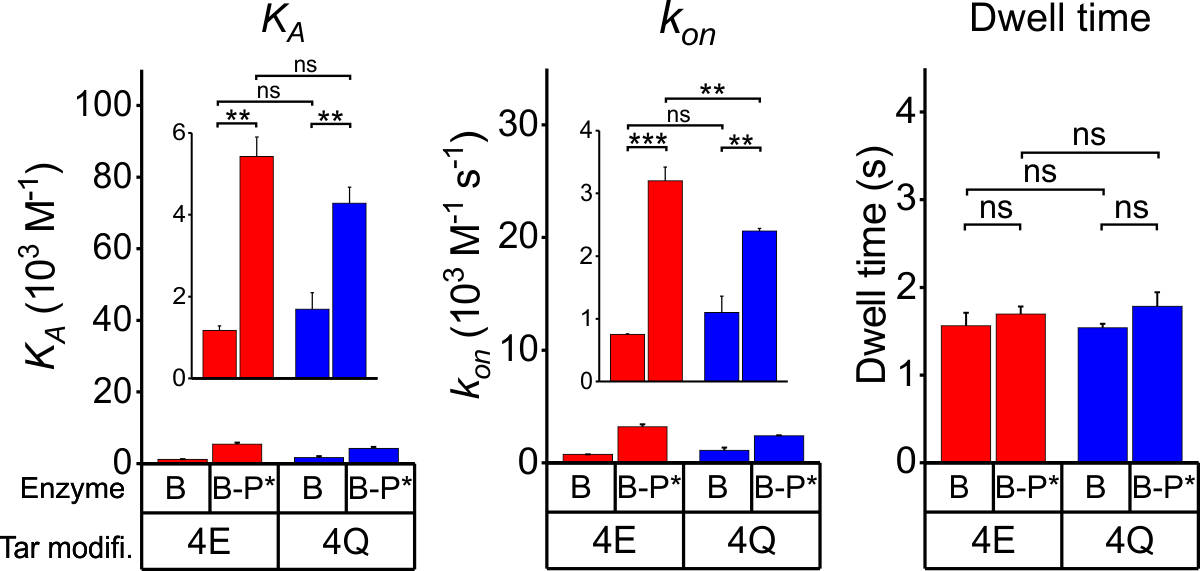

Supplement: FIG S2 [file mbio.03106-21-sf002.tif]

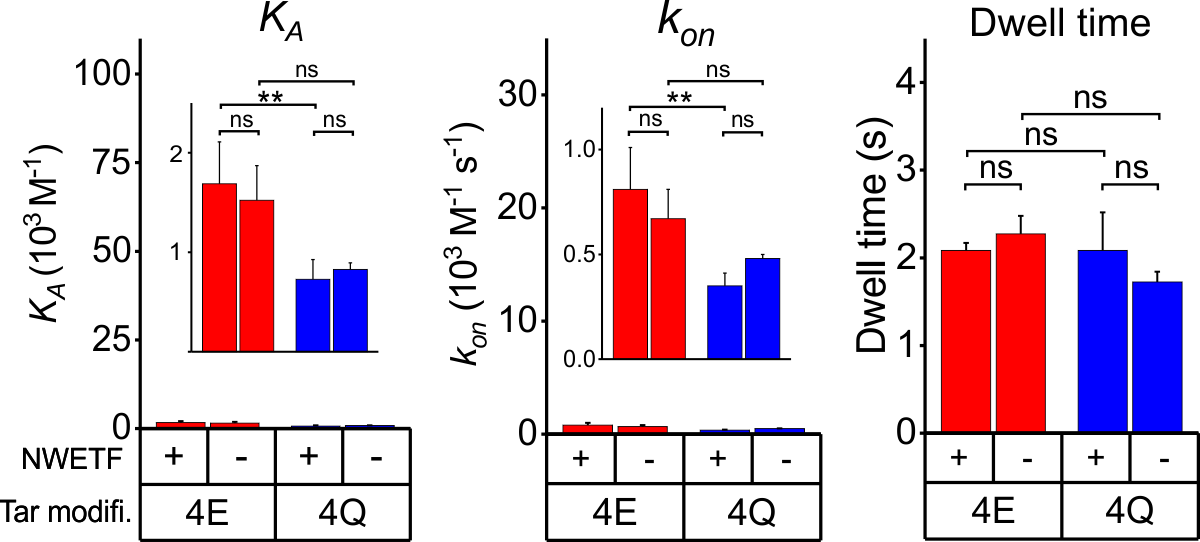

Supplement: FIG S3 [file mbio.03106-21-sf003.tif]

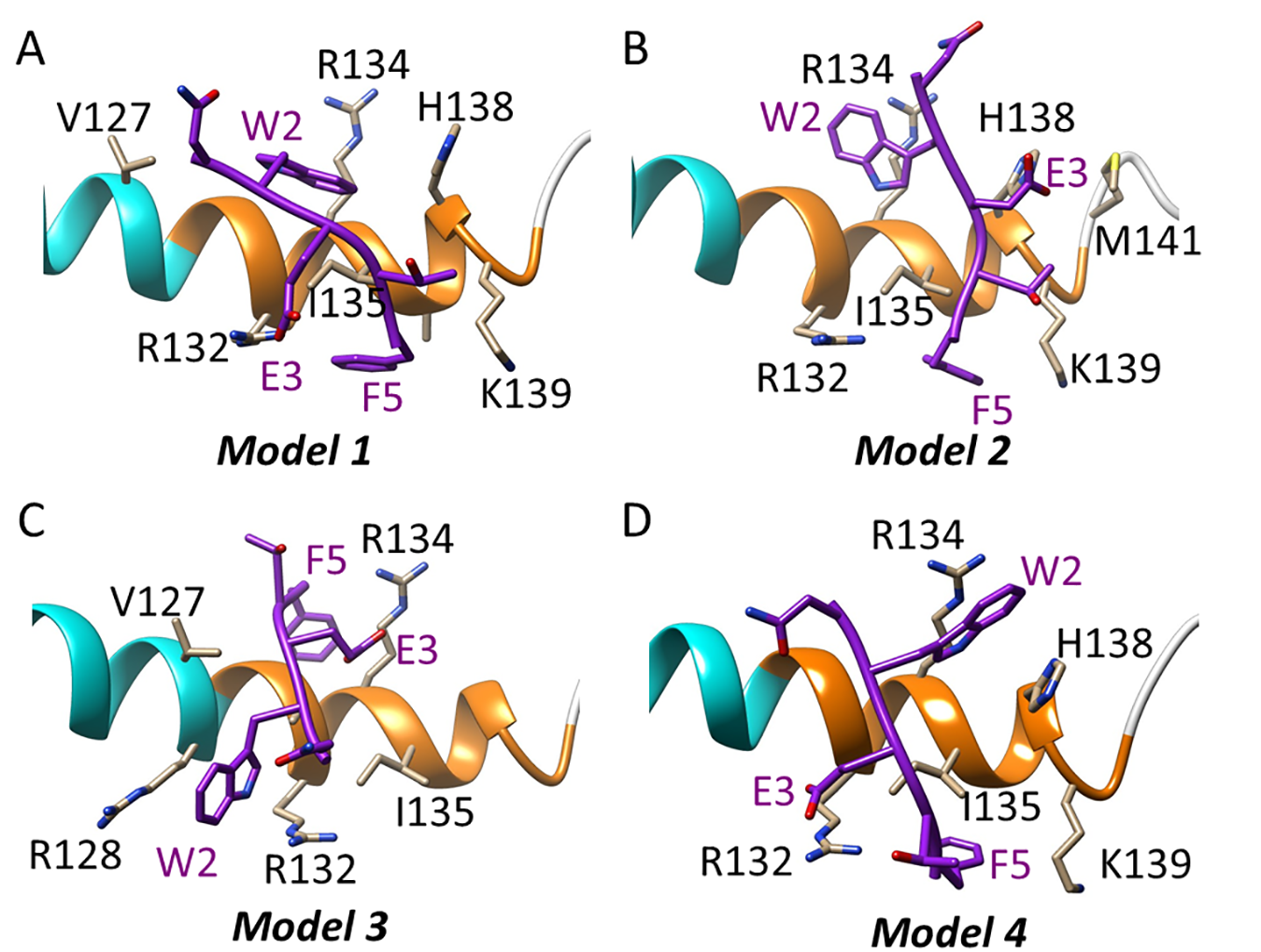

Supplement: FIG S4 [file mbio.03106-21-sf004.tif]

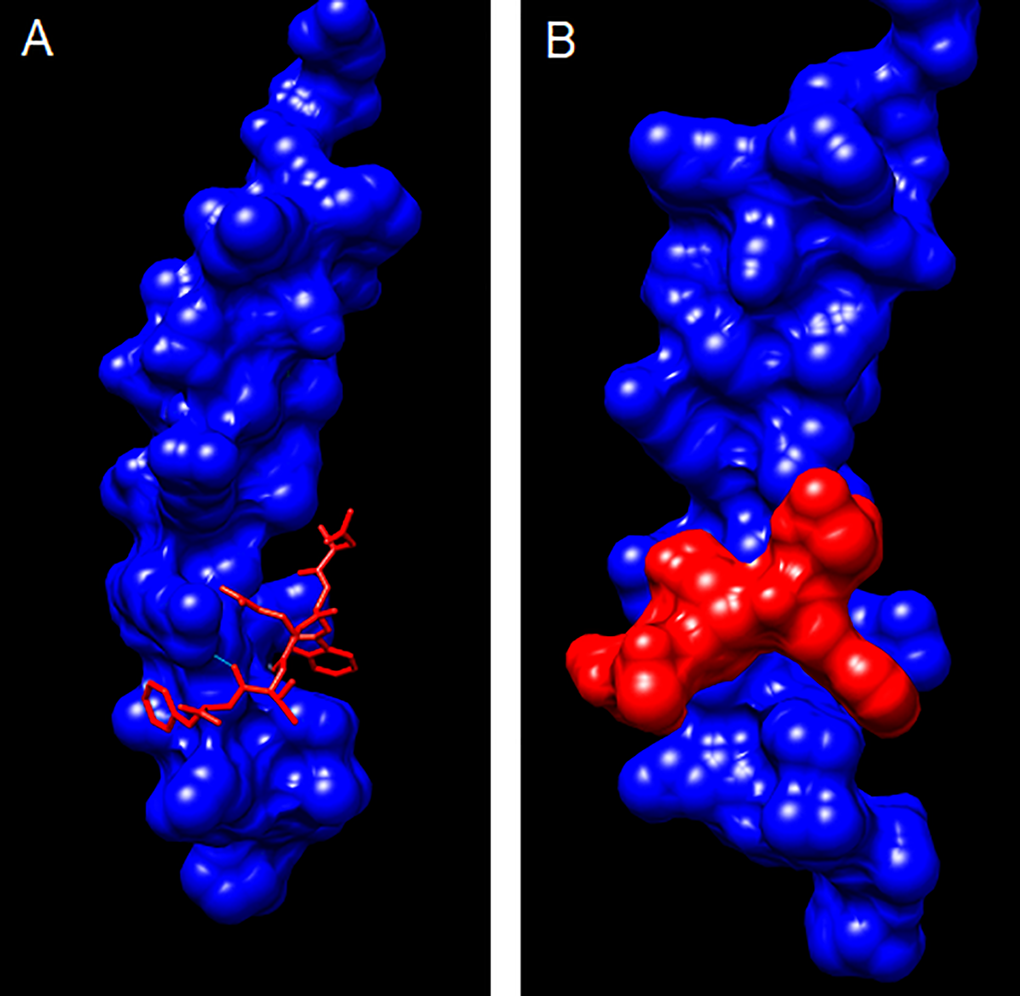

Supplement: FIG S5 [file mbio.03106-21-sf005.tif]

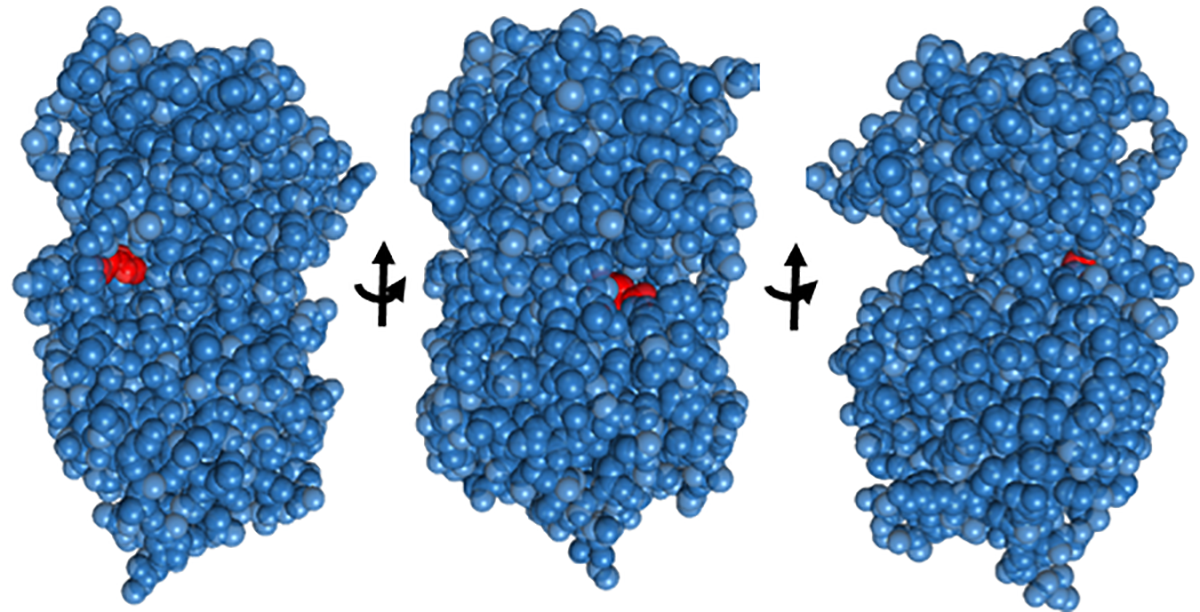

Supplement: FIG S6 [file mbio.03106-21-sf006.tif]
