# Supplementary material for: A Selective Tether Recruits Activated Response Regulator CheB to Its Chemoreceptor Substrate
Source: mBio. 2021 Nov 23;12(6):e03106-21. doi: 10.1128/mBio.03106-21 (PMC8609364; doi:10.1128/mBio.03106-21)

Table S1. Alignment of non-redundant CheB sequences from bacterial species containing 1 CheB and ≥1 chemoreceptor with a carboxyl-terminal pentapeptide NWETF or NW


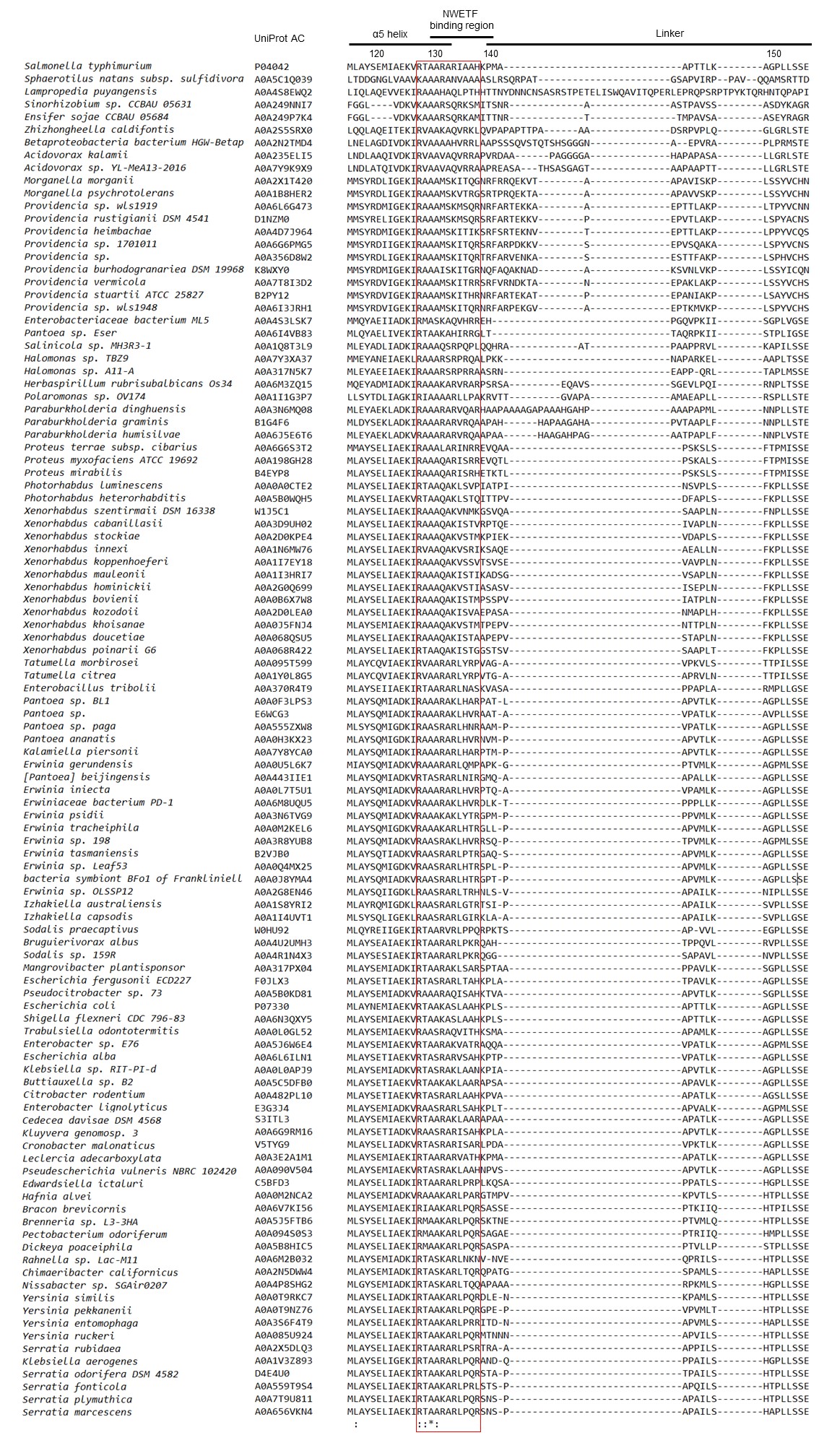

Supplement: TABLE S1 [file mbio.03106-21-st001.docx]
